# Somatotopic Map and Inter- and Intra-Digit Distance in Brodmann Area 2 by Pressure Stimulation

**DOI:** 10.1038/srep30243

**Published:** 2016-07-25

**Authors:** Mi-Hyun Choi, Sung-Phil Kim, Hyung-Sik Kim, Seon-Young Gim, Woo-Ram Kim, Kyung-Ryul Mun, Dae-Woon Lim, Bongsoo Lee, Soon-Cheol Chung

**Affiliations:** 1 Department of Biomedical Engineering, Research Institute of Biomedical Engineering, College of Biomedical & Health Science, Konkuk University, Chungju, South Korea; 2 Department of Human and Systems Engineering, Ulsan National Institute of Science and Technology, Ulsan, South Korea; 3 Department of Information and Communication Engineering, Dongguk University, Seoul, South Korea; 4 School of Energy Systems Engineering, Chung-Ang University, Heukseok-dong, Dongjak-gu, Seoul, South Korea

## Abstract

The somatotopic representation of the tactile stimulation on the finger in the brain is an essential part of understanding the human somatosensory system as well as rehabilitation and other clinical therapies. Many studies have used vibrotactile stimulations and reported finger somatotopic representations in the Brodmann area 3 (BA 3). On the contrary, few studies investigated finger somatotopic representation using pressure stimulations. Therefore, the present study aimed to find a comprehensive somatotopic representation (somatotopic map and inter- and intra-digit distance) within BA 2 of humans that could describe tactile stimulations on different joints across the fingers by applying pressure stimulation to three joints-the first (p1), second (p2), and third (p3) joints-of four fingers (index, middle, ring, and little finger). Significant differences were observed in the inter-digit distance between the first joints (p1) of the index and little fingers, and between the third joints (p3) of the index and little fingers. In addition, a significant difference was observed in the intra-digit distance between p1 and p3 of the little finger. This study suggests that a somatotopic map and inter- and intra-digit distance could be found in BA 2 in response to pressure stimulation on finger joints.

Recent advances in haptic technology have drawn attention to the brain mechanisms underlying the perception of tactile stimuli and the processing of tactile information. One of the basic approaches for understanding tactile processing in the brain is the study of how humans sense objects with their fingers, as the fingers are a primary tactile sensor and occupy the biggest area in the primary somatosensory cortex (S1). In particular, many neuroimaging studies have revealed the somatotopic representations of tactile sensation of the fingers^1^,^2^,^3^,^4^,^5^,^6^,^7^,^8^,^9^,^10^,^11^,^12^,^13^,^14^. These representations could create a somatotopic map of different stimulation locations over the fingers onto the corresponding areas in S1. Such maps could also provide information about the somatotopic distances between activated areas, which depict how stimulation locations on the fingers are relatively represented in S1. A somatotopic distance can be further specified as an inter-digit distance and an intra-digit distance, where an inter-digit distance is the distance between activated areas in S1 in response to tactile stimuli provided to different fingers, and an intra-digit distance is the distance in response to tactile stimuli provided to different joints in a single finger^4^,^5^,^6^,^7^,^9^,^10^,^11^,^12^,^13^,^14^. The somatotopic map and distance information can be useful for clinical studies. For instance, one can use the map and distances to monitor or induce plastic changes in specific S1 areas through the rehabilitation of affected fingers in patients with stroke or focal hand dystonia.

Numerous studies have examined the somatotopic maps and inter- and intra-digit distances in S1 induced by various tactile stimuli using functional magnetic resonance imaging (fMRI)^1^,^2^,^3^,^4^,^5^,^6^,^7^,^8^,^9^,^10^,^11^,^12^,^13^,^14^. Most studies have revealed a somatotopic map in BA 3 by stimulating fingers using various means such as piezoelectric (50 Hz or lower), brush, electric, and air^1^,^2^,^3^,^4^,^5^,^6^,^7^,^9^,^10^,^11^,^12^,^13^,^14^. The inter-digit distances were also estimated from the somatotopic maps in BA 3 when various tactile stimuli were applied to the first joint of each finger^1^,^2^,^4^,^9^,^11^,^13^. In addition, somatotopic representations of within-finger stimulations in BA 3 have been reported when electric stimulation was applied to each joint of the middle finger^10^ or when air stimulus was provided to the thumb, index, and ring fingers at intervals of 1.5 cm^12^. Schweisfurth *et al*. have recently estimated intra-digit distances in BA 3 by applying 32-Hz vibrotactile stimulation to the all joints of the five fingers^14^.

However, comprehensive somatotopic representations of tactile sensation at all the joints over the fingers remain to be further explored. Although the previous studies revealed somatotopic maps and inter-digit distances in BA 3, they were estimated only on the basis of the stimulations applied to the first joint of each finger; the other joints were not analyzed. Further, the previous studies constructed somatotopic maps and inter- and intra-digit distances in BA 3 mainly by introducing vibration stimulations. On the other hand, somatotopic representations in S1 in response to pressure stimulations on the fingers have not been published. An electrophysiological study has shown that BA 3 is activated by most tactile stimulations such as vibration, pressure, and others; BA 2 by pressure stimulation, joint position, and complex touch; and BA 1 by vibration stimulation^15^. Accordingly, BA 2 as well as BA 3 can be sensitive to pressure stimulations and thus become an area where somatotopic representations for pressure on the fingers may be found. However, little is known about somatotopic representations in BA 2. Finally, simultaneous estimation of inter- and intra-digit distances in BA 2 from an integrated somatotopic map is not explored. Therefore, the present study aims to integrate the inter-, and intra-digit distances in a unified way by defining an inter/intra-digit distance. In this study, the inter-, or intra-digit distance is defined as the Euclidean distance between the 3D coordinates of activation locations in BA 2 (defined in the Montreal Neurological Institute (MNI) space) in response to the stimulations of the same joints of two different fingers or two different joints of the same finger, respectively. On the other hand, the inter/intra-digit distance is defined as the Euclidean distance between the 3D activation coordinates within BA 2 of a reference location and those of all other joints of all the fingers, where the activation location for the p1 of the index finger is set as the reference in this study. With this definition, we measure all the distances of each joint of each finger from the reference location (p1 of the index finger) all at once from which we can examine whether it is plausible to distinguish activation locations for each joint of each finger within BA 2. Such estimation may provide a comprehensive view of the somatotopic representation of inter- and intra-digit tactile stimulations.

The present study, therefore, aims to find a comprehensive somatotopic representation of the fingers by providing a pressure stimulus to the three joints [first (p1), second (p2), and third (p3) joints] of the four fingers (index, middle, ring, and little fingers) and detecting activation locations in BA 2 of humans using fMRI. By identifying a peak activation location (peak coordinates) among the activation locations for each joint of each finger, we construct a somatotopic map and estimate the inter- and intra-digit distances to discriminate tactile stimulations between different joints as well as different fingers.

## Methods

### Subjects

Ten healthy male (mean ± S.D.: 26.6 ± 2.5 years old) college students participated in the study. None of the participants reported having a history of psychiatric, physiological, or neurological disorders. The overall procedure was explained to all participants, who in turn gave their consent for the procedure. Written informed consent was obtained from all participants prior to the experiment. All experimental procedures were approved by and performed under the regulations of the Institutional Review Committee of Korea University (KU-IRB-11-46-A-1).

### Pressure stimulation

**Figure 1:**
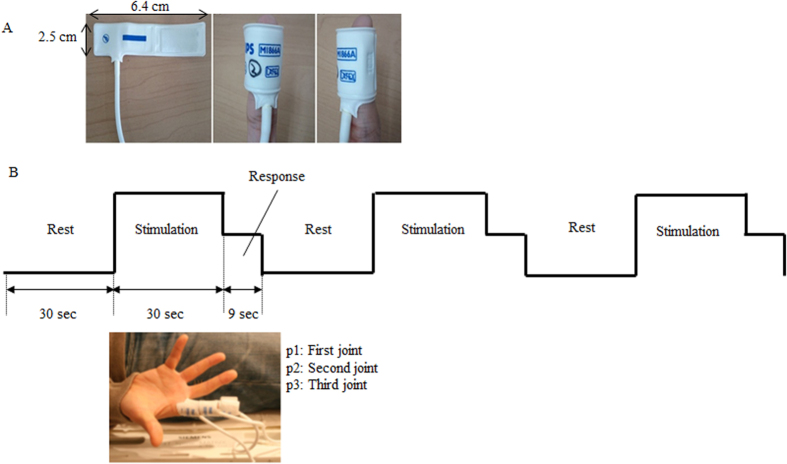
(**A**) An MR-compatible pressure stimulator, built by using a non-invasive blood-pressure measurement cuff for infants, was used to provide a pressure stimulus to each joint of the fingers of the right hand by the injection of air into the cuff. The cuff size was 6.4 cm × 2.5 cm. (**B**) The experimental paradigm and a subject lying in the scanner with three pressure stimulators attached to each joint of the index finger. During the rest phase, the subjects rested with their eyes closed without pressure stimulation for 30 s. During the stimulation phase, a pressure stimulus was provided to one of the three joints for 30 s. During the response phase, the subjects pressed one of the three buttons with their left hand, corresponding to the location of the stimulated joint. The responses were collected to confirm whether the subjects correctly sensed the location of a given pressure stimulus. The first, second, and third joints are denoted p1, p2, and p3, respectively.

Pressure stimulation was applied using an MR-compatible pressure stimulator developed by our research team^16^. Pressure was applied to each finger joint by injecting air into a cuff (M1866A, Philips, Netherlands) used for non-invasive blood-pressure measurement of an infant (Fig. 1A). Pneumatic inflation, regulation, and deflation were performed by controlling the rolling pump and solenoid valve with a transistor switch after generating pneumatic pressure by employing the rolling pump used in a commercial blood-pressure manometer. The pneumatic pressure generated by the pump was transmitted to the cuff using a 7-m air tube, and the cuff size was 6.4 cm × 2.5 cm (Fig. 1A). E-Prime S/W (Psychology Software Tools, Inc.) was used to control the stimulation parameters such as pressure strength, exertion time, etc. Synchronization of the MR scanner and MR-compatible pressure stimulator was performed using a trigger signal generated from the LPT-1 printer port of a PC where the E-Prime software was installed^16^. The brain-function image was acquired by applying pressure stimulation with a certain strength (8.5 psi) to the three joints [first (p1), second (p2), and third (p3) joints] of the four fingers (index, middle, ring, and little fingers) in the right hand.

### Experimental design

Three pressure stimulators (cuffs) were attached to the three joints [first (p1), second (p2), and third joints (p3)] of a randomly selected finger (index, middle, ring, or little finger) (Fig. 1B). The session consisted of three blocks, and each block consisted of a rest phase (30 s), a stimulation phase (30 s), and a response phase (9 s). The rest phase, in which no pressure stimulation was applied, was defined as the state where the subject comfortably lay down for 30 s with his eyes closed. In the stimulation phase, pressure stimulation was randomly applied for 30 s to one of the three joints (p1, p2, or p3) of the selected finger (index, middle, ring, or little finger). Because a session had three stimulation phases, pressure stimulation could be applied to all three joints. In the response phase, a response button was installed on the left hand of the subjects to examine whether or not they accurately recognized the location of the applied pressure stimulation, and they were instructed to push the number of the button that corresponded to the pressure stimulation among the three joints (p1, p2, or p3) according to their perception. Three repeated experiments were conducted for a selected finger in each session. Therefore, pressure stimulation was applied three times to each of the three joints of a selected finger during the three sessions. The experiments were also performed on the other three fingers following the same procedure described earlier. All the subjects participated in a total of 12 sessions for the three repeated experiments conducted for each finger (three sessions/finger × four fingers = 12 sessions). Counterbalancing was conducted for the stimulation order on each finger and joint across the subjects. Between the successive sessions, the subjects took a 5-min break while comfortably lying in the scanner with no task. Considering the fatigue and the attenuation of task engagement in the subjects due to the long experimental time, we allowed the subjects to step out of the scanner and take a break after six sessions and resumed the remaining sessions after 30 min.

All the subjects wore a headset to block auditory and visual stimulations and closed their eyes during the experiment. They were instructed to minimize the movement of their hands and heads. During functional imaging, the subjects lay on the MR table in a supine position holding their right arm from the elbow down in a padded cast that supported the dorsal part of the hand. They were instructed to rest their arm against the magnet bore so that both the arm and the hand were relaxed. To ensure that the hand and finger positions were maintained as consistently as possible across the subjects, each subject was instructed on how to maintain their hand and finger positions and practiced positioning with the air cuffs attached to the fingers before performing the fMRI experiment.

By investigating the response acquired from the response phase, we analyzed the brain-function images of the eight subjects (mean ± S.D.: 25.4 ± 1.3 years old) among the ten who recognized the location of pressure stimulation as its target with 100% accuracy.

### Image acquisition

The images were scanned by a 3T MRI system (Magnetom TrioTim, Siemens Medical Systems, Erlangen, Germany) using a standard 32-channel head coil. Anatomical images were obtained using a T1-weighted 3D MPRAGE sequence with repetition time TR = 1900 ms, echo time TE = 2.48 ms, flip angle = 9°, field of view FOV = 200 mm, and spatial resolution = 0.8 × 0.8 × 1 mm^3^. Functional images were obtained using a T2*-weighted gradient echo-planar imaging sequence with TR = 3000 ms, TE = 30 ms, flip angle = 90°, FOV = 192 mm, slice thickness = 2 mm, and in-plane resolution = 1.5 × 1.5 mm^2^.

### fMRI data analysis

The fMRI data were analyzed using SPM 8 (Wellcome Department of Cognitive Neurology, London, UK). All functional images were aligned with the anatomic images of the study using affine transformation routines built into SPM 8. The realigned scans were co-registered with the anatomic images of the participants obtained in each session and normalized to the SPM8 template image that uses the space defined by the Montreal Neurological Institute. Motion correction was performed using sinc interpolation. The time-series data were filtered using a 240-s high-pass filter to remove artifacts due to cardiorespiratory and other cyclical influences. The functional map was smoothened using a 3-mm isotropic Gaussian kernel prior to the statistical analysis. The statistical analysis was performed both individually and as a group using the general linear model and the theory of Gaussian random fields implemented in SPM 8. The statistical parametric maps with t-statistics were computed. Analysis of the individual subjects was performed at a significance threshold of *p* < 0.05, corrected for multiple comparisons. We used random effect analysis to compute the group activation.

We analyzed the activation of the somatosensory cortical area in the left hemisphere contralateral to the stimulated right hand. In particular, we examined the activation in BA 2 using the region-of-interest (ROI) method on the basis of our hypothesis that pressure stimulations would activate neural responses in BA 2^15^. Using the subtraction method (stimulation phase−rest phase), the activated voxels were sought in BA 2 in response to the stimulation of each joint and finger. Among the activated voxels in BA 2 for each joint, we selected the one with the peak activation (measured by the highest t-value). The anatomical location of that voxel, represented by the x-, y-, and z-coordinates in the Montreal Neurological Institute (MNI) space, was defined as a peak coordinate for each joint and finger. Here, the x-coordinate is a given number of pixels along the lateral–medial axis, the y-coordinate is a given number of pixels along the anterior–posterior axis, and the z-coordinate is a given number of pixels along the inferior–superior axis. For each subject, we identified the peak coordinates of a total of 12 joints.

### Measurement of inter-, intra-, and inter/intra-digit distances

From the measurements of the inter-digit and intra-digit distances used in the previous studies^1^,^2^,^4^,^5^,^6^,^7^,^9^,^10^,^11^,^12^,^13^,^14^, we calculated the inter-digit and intra-digit distances as described later. In addition, we also calculated the inter/intra-digit distances in the same manner. We first constructed difference vectors between the fingers and joints using the 3D peak coordinates^1^,^2^,^4^,^7^,^9^ to normalize individual peak coordinate data and to estimate the spatial direction of the somatotopic representations of the pressure stimulations across the fingers and joints. Using the difference vectors, we then calculated the Euclidean distance (E.D.) between the joints to estimate how much the somatotopic representations of the pressure stimulations of each joint are separated. More details of the calculation of the inter-, intra-, and inter/intra-digit distances follow.

First, the inter-digit distance calculated by the difference vector measured the distance from the peak coordinates at BA 2, which was activated by the introduction of pressure stimulation on each joint (p1, p2, or p3) of the index finger to the peak coordinates at BA 2 activated by the exertion of the pressure stimulation on the same joints of the middle, ring, and little fingers. For instance, the difference vectors of the inter digit at p1 was calculated by subtracting the x-, y-, and z-coordinates of the peak coordinates activated by the pressure stimulation of p1 in the middle, ring, and little fingers from the x-, y-, and z-coordinates of the peak coordinates activated by the pressure stimulation on p1 of the index finger [Eq. 1(a)]. The difference vector of the inter digit was calculated in the same manner using p2 and p3 of the index finger as the reference.


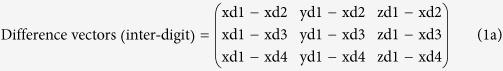


xd1, yd1, and zd1 represent the peak coordinates of the index finger; xd2, yd2, and zd2 represent those of the middle finger; xd3, yd3, and zd3 represent those of the ring finger; and xd4, yd4, and zd4 represent those of the little finger in the three directions x, y, and z.

Second, the intra-digit distance calculated using the difference vector measured the distance to the peak coordinates at BA 2 activated by the pressure stimulation of p2 and p3 with the peak coordinates at BA 2 activated by the pressure stimulation of p1 in each finger as the reference. For instance, the difference vectors of the intra digit in the index finger was calculated by subtracting the x-, y-, and z-coordinates of the peak coordinates activated by the pressure stimulation of p2 and p3 from the x-, y-, and z-coordinates of the peak coordinates activated by the pressure stimulation of p1 as its reference [Eq. 1(b)]. The difference vector of the middle, ring, and little fingers was calculated using Eq. 1(b):


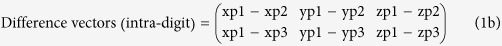


xp1, yp1, and zp1 represent the peak coordinates of the first joint; xp2, yp2, and zp2 represent those of the second joint; and xp3, yp3, and zp3 represent those of the third joint in the three directions x, y, and z.

Third, the inter/intra-digit distance measures distances from the peak coordinate at BA 2 activated by the pressure stimulation on p1 of the index finger to the peak coordinates at BA 2 activated by the pressure stimulation on all other joints. This distance is different from the inter-digit distance, which measures a distance between the same joints of different fingers, or the intra-digit distance, which measures a distance between different joints within the same finger. Since the inter-digit distance or intra-digit distance alone does not include a distance between different joints of different fingers (e.g. between p1 of the index finger and p2 of the middle finger), we employ the inter/intra-digit distance to incorporate distances between any joints across the fingers. Specifically, by measuring a distance of every joint to p1 of the index finger, we aim to examine how widely the peak coordinates activated by the pressure stimulation on any joint from p1 of the index finger up to p3 of the little finger are distributed at BA 2. Also, the inter/intra-digit distance can provide information of distances from p1 of the index finger to every joint within the middle, ring or little finger. For instance, we can examine how similar distances of the peak coordinates of p1, p2 and p3 of the middle finger from that of p1 of the index finger are to each other. Equation 1(c) was similarly calculated by subtracting the x-, y-, and z-coordinate values of the peak coordinates for all other joints from the x-, y-, and z-coordinate values of the peak coordinates activated by the pressure stimulation on p1 of the index finger as its reference.


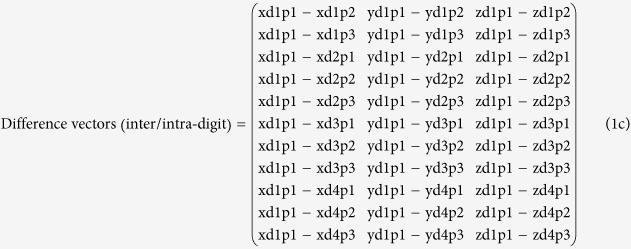


xd1p1, yd1p1, and zd1p1 represent the peak coordinates of the first joint; xd1p2, yd1p2, and zd1p2 represent those of the second joint; and xd1p3, yd1p3, and zd1p3 represent those of the third joint in the three directions x, y, and z of the index finger. d2, d3, and d4 represent the middle, ring, and little fingers, respectively.

Finally, the E.D. was calculated as the square root of the sum of the squares of each element in the difference vector. For instance, Eq. (2) is the square root of the sum of the squares of an element of the difference vector [Eq. 1(b)] to acquire the E.D. between p1 and p2 at the intra digit. In other words, the E.D. of the inter digit, intra digit, and inter/intra digit were respectively calculated by the square roots of the sum of the squares of each element in Eqs 1(a–c).


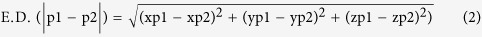


We performed the Wilcoxon matched paired t-test (PASW 18.0) to validate the statistical significance of the inter-, intra-, and inter-/intra-digit difference vectors and Euclidean distances to discriminate tactile stimulations between different joints and different fingers within Brodmann area 2 (BA 2).

## Results

When pressure stimulation was applied to the ring finger, activation in BA 2 was observed in only three out of the eight subjects. Because it was not considered a significant result, the stimulation on the ring finger was excluded from the analysis.

**Figure 2:**
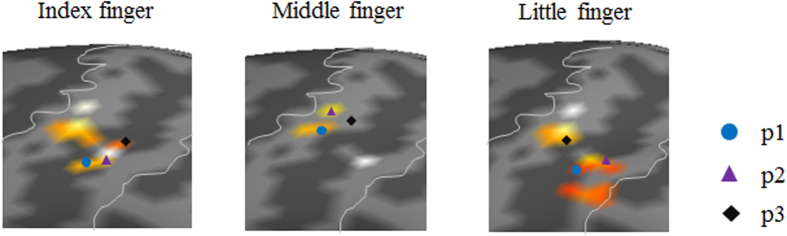
The group analysis result of the activation in Brodmann area 2 (BA 2) in response to pressure stimuli on individual joints and fingers. The three markers for each finger represent a peak coordinate of the activation for each joint: p1 (circle), p2 (upward triangle), and p3 (diamond). The peak coordinate is defined as a 3D coordinate (mm) of the Montreal Neurological Institute (MNI) space where the activation level peaked (evaluated by the t-value) for each stimulus. Note that no significant somatotopic map was obtained for the stimulation of the ring finger.

**Figure 3:**
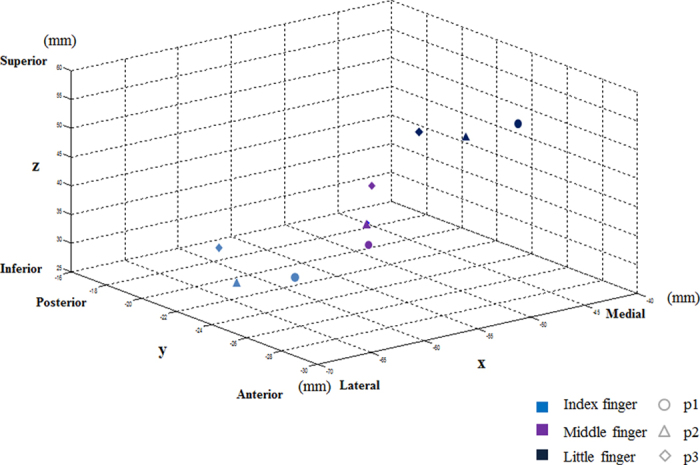
The illustration of the peak coordinates in Brodmann area (BA 2) in response to pressure stimulations of each joint in three fingers (index, middle, and little fingers). The peak coordinates (mm) are represented in the 3D MNI (Montreal Neurological Institute) space composed of three axes: in the lateral–medial (x-axis), the anterior–posterior (y-axis), and the inferior–superior (z-axis) directions. Following the sequence of the fingers {index, middle, little}, the peak coordinates varied in the direction from lateral to medial and from inferior to superior for all the joints. Following the sequence of the joints, {p1, p2, p3}, the peak coordinates varied in the direction from anterior to posterior for all the fingers. Each marker depicts the peak coordinate of each joint: p1 (circle), p2 (upward triangle), and p3 (diamond). Different fingers are marked by different colors: light blue (index), purple (middle), and black (little).

The group analysis result for the peak coordinates at BA 2 activated for each joint of the index, middle, and little fingers upon application of the pressure stimulation is shown in Table 1 (MNI coordinates). Figure 2 illustrates the MNI peak coordinates (Table 1) of each joint with different colors to indicate the activation locations of three different joints within each finger. Also, Fig. 3 illustrates the 3D MNI peak coordinates of every joint of the index, middle, and little fingers with different shapes (for joints) and colors (for fingers) on the axes of x (lateral-medial), y (anterior-posterior), and z (inferior-superior) so as to indicate topological directions of the activation locations. Figure 3 shows that the peak coordinates at BA 2 were observed from the lateral to the medial direction and from the inferior to the superior direction following the sequence of the index, middle, and little fingers for all joints. In addition, the peak coordinates at BA 2 were observed from the anterior to the posterior direction following the p1, p2, and p3 sequence in all fingers.

The average values of the difference vector of the inter-, intra-, and inter/intra-digits and the E.D. of the eight subjects are presented in Table 2. No statistically significant difference was observed for the inter-, intra-, and inter/intra-digit distance calculated by the difference vector.

First, we conducted a statistical analysis on the inter-digit distances to assess whether the activation locations for different fingers of same joint were significantly different within BA 2. The distance between the index and little fingers was 27.3 ± 0.9 mm for p1 (p = 0.036), and 22.0 ± 1.6 mm (p = 0.047) for p3, showing a significant difference in the corresponding activation locations. No significant difference was observed in the E.D. of each finger for p2.

Second, we conducted a statistical analysis on the intra-digit distances to assess whether the activation locations for different joints of the same finger were significantly different within BA 2. The distance between p1 and p3 of the little finger was 10.0 ± 0.8 mm (p = 0.029), which showed a significant difference in the corresponding activation locations.

Third, we conducted a statistical analysis on the inter/intra-digit distances to assess whether the activation locations for each joint of each finger were significantly different within BA 2, with reference to p1 of the index finger. The distance between p1 of the index and little fingers was 27.3 ± 0.9 mm (p = 0.036), which showed a significant difference.

## Discussion

Pressure stimulation was applied on each joint [first (p1), second (p2), or third (p3) joint] of the four fingers (index, middle, ring, and little fingers), and the peak coordinates were extracted from BA 2 using fMRI. We examined whether we could distinguish activation locations (represented by peak coordinates) within BA 2 in response to stimulations of each joint of each finger by building a somatotopic map and measuring inter-, intra-, and inter/intra-digit distances.

When pressure stimulation was applied to the four fingers, no distinctive activation was observed in the ring finger compared with the other fingers. Therefore, a somatotopic map was constructed on the basis of the peak coordinates at BA 2 in relation to each joint of the three fingers excluding the ring finger, and the inter-, intra-, and inter/intra-digit distances (E.D.) were calculated.

Although all eight subjects whose fMRI data were analyzed in this study reportedly discriminated the location of a stimulated joint in all the fingers, including the ring finger, without an error, we observed the activation of BA 2 in the contralateral S1 only in a few subjects when we stimulated the ring finger. The previous studies by Hegner and colleagues reported dominant activation in the right S1 (ipsilateral to the stimulated hand) when subjects discriminated different tactile stimuli with the right hand^17^,^18^. Other studies observed more activation in the secondary somatosensory cortex (S2) than in S1 when people performed tactile discrimination tasks^19^,^20^,^21^,^22^. Weinstein reported that the sensitivity of the ring finger was relatively lower than that of the other fingers when the tactile sensitivity of all the five fingers was compared^23^. From the foregoing findings, we speculate that the subjects might be able to perceive the ring finger stimulation with neural responses over contralateral BA 2 as well as ipsilateral S1 and S2, but the low sensitivity of the ring finger might induce less activation of contralateral BA 2 than other fingers, which would make it relatively more difficult to detect the activations using indirect fMRI measurement. However, it is still unclear from the results of our study why we did not observe activation in contralateral S1 for the ring finger; more in-depth psychophysical and neuroimaging studies are required that examine the involvement of neural networks over S1 and S2 in the perception of tactile stimuli on individual fingers.

In previous studies, a somatotopic map at BA 3 was constructed by introducing a vibration of 50 Hz or by applying lower, brush, electric, and air-puff stimulation to the first joint of the five fingers^1^,^2^,^3^,^4^,^5^,^6^,^7^,^9^,^10^,^11^,^12^,^13^,^14^. As a result, activation of BA 3 was reported to be observed from the lateral to the medial direction, from the inferior to the superior direction, and from the anterior to the posterior direction following the sequence of the thumb to the little finger in the case of the first joint^4^,^9^,^11^,^13^. Apart from the first joint of each finger, the pressure stimulation was also applied to the second and third joints in this study, which was not applied in the preceding studies. Rather than focusing on BA 3, the result was obtained by focusing on BA 2, the area that sensitively responds to pressure stimulation. As a result, a somatotopic map of BA 2 was constructed from the lateral to the medial direction and from the inferior to the superior direction in all joints following the sequence of the index, middle, and little fingers. Although the presented method of stimulation and observed activation area (BA 3 versus BA 2) differed between the previous and the present studies, the pattern of the somatotopic map by tactile stimulation was shown to be similar. However, no clear trend from the anterior to the posterior direction was observed in the present study.

In most previous studies, the inter-digit distance was calculated using the location of BA 3 activated by applying vibration, electric, and brush stimulation to the first joint in a finger^2^,^4^,^9^,^11^. With regard to the inter-digit distance at BA 3 involving the first joint of the five fingers, the distance from the index finger was approximately 5.7–10.6 mm, that from the middle finger was approximately 5.4–11.2 mm, that from the ring finger was approximately 4.7–16 mm, and that from the little finger was approximately 3.7–17 mm using the thumb finger as the refs 2,4,9 and 11. In our study, the distance between the index and little fingers was 27.3 mm, which showed a significant difference for the inter-digit distance at BA 2 relative to the first joint in each finger. Further, concerning the inter-digit distance at BA 2 with regard to the third joint of the index and little fingers, the distance was 22.0 mm, which is a significant difference. The inter-digit distance at BA 2 was observed to be relatively greater compared with that at BA 3 observed in the previous studies. This result is due to the difference in the anatomical size between BA 3 and BA 2. In general, the size of BA 2 is greater than that of BA 3^24^, and the larger inter-digit distance of BA 2 of the joints in each finger is attributable to this reason. In addition, the discrepancy may result from the different stimulation methods such as the type, strength, and other factors used in the previous studies. Weinstein (1962) applied pressure stimulation with varying strength on each joint of the five fingers, and the tactile sensitivity result was reported using the subjective evaluation of the participants. The sensitivity of the first joint (p1) was reported to be the highest, followed by the third (p3) and the second (p2) joints. In addition, the index and little fingers were reported to display high tactile sensitivity compared with the other fingers because of the high distribution of cutaneous receptors and the wide distribution of the neurons corresponding to it compared with the other fingers^7^,^25^,^26^. Therefore, we determine that a significant difference in the inter-digit distance exists between the index and little fingers in the p1 and p3 joints owing to such tactile sensitivity.

Although many studies regarding the somatotopic map and the inter-digit distance on the first joint of the finger have been conducted, studies on the somatotopic map and the intra-digit distance relative to each joint of other fingers have been lacking. However, Schweisfurth *et al*.^14^. have recently reported that a somatotopic map was observed at BA 3 from the anterior to the posterior direction and from the lateral to the medial direction following the sequence from p1 to p3 for each joint in the fingers when a 32-Hz vibration stimulation was introduced to the all joints of the five fingers. In this study, we observed that the somatotopic map is displayed at BA 2 from the anterior to the posterior direction in the sequence from p1 to p3 for each finger when pressure stimulation is applied. The somatotopic map patterns due to tactile stimulation were observed to be similar, although the applied stimulation method and observed activation area in this study were different from those of the previous studies. However, no distinctive trend from the lateral to the medial direction was observed in the present study. In conclusion, we observed that the somatotopic map at BA 3 and BA 2 showed a similar pattern, although different tactile stimulations were applied on each joint in the finger.

Schweisfurth *et al*.^14^. reported the result of the intra-digit distance at BA 3. In the case of the index finger, the distance from p1 to p2 was 4 mm, and that from p1 to p3 was 6.2 mm. The distance from p1 to p2 was 2.9 mm, and that from p1 to p3 was 6.6 mm for the middle finger. The distance from p1 to p2 was 2.9 mm, and that from p1 to p3 was 5 mm for the ring finger. Further, the distance from p1 to p2 was 2.3 mm, and that from p1 to p3 was 3.4 mm for the little finger. However, statistical significance was not observed in any of the cases. The result of the present study indicates that the distance of the index finger from p1 to p2 was 4.3 mm, and that from p1 to p3 was 7.7 mm. The distance of the middle finger from p1 to p2 was 12.2 mm, and that from p1 to p3 was 10.2 mm. The distance of the little finger from p1 to p2 was 9.8 mm, and that from p1 to p3 was 10.0 mm. Among these cases, a significant difference in the intra-digit distance at BA 2 was observed only between p1 and p3 of the little finger. Overall, the intra-digit distance at BA 2 observed in this study was relatively greater than the intra-digit distance at BA 3 observed in previous studies. We mentioned in the discussion on the inter-digit distance that the difference in the anatomical size of BA 2, the stimulation method, and other factors may have caused the difference. We also found that a relatively clear significant difference was observed in this study for the intra-digit distance between p1 and p3 only in the little finger compared with the other fingers owing to the aforementioned tactile sensitivity.

The inter/intra-digit distance at BA 2 for each finger and joint activated by stimulation was calculated in this study using p1 of the index finger as the reference. Overall, an increasing trend was observed in the distance from the index to the little finger, and the distance between p1 of the index and the little fingers was 27.3 mm, which showed a significant difference.

In conclusion, pressure stimulation was applied on each of the three joints in the three fingers in this study, and a somatotopic map was constructed for each finger and joint using the activation location at BA 2, which was reported to most sensitively respond to pressure stimulation among BA 1, BA 2, and BA 3 of the somatosensory area. The activation distance was then measured for comparison. The present study showed that a somatotopic map similar to that in previous studies under vibration stimulation could be constructed at BA 2 using pressure stimulation and the activation distance corresponding to the level of each finger and joint. The result of this study, which comprehensively analyzed the somatotopic map and inter- and intra-digit distance according to the pressure stimulation, can be used as basic data to construct an accurate somatotopic map of the fingers.

Although it was not observed in this study, the activation locations of BA 1 and BA 3 (in addition to BA 2) were identified; however, activation was observed in only two out of the eight subjects. In other words, the activation of each finger and joint was distinctive in BA 2, which dominantly receives information about tactile pressure stimulation, but little activation was observed in BA 1 and BA 3. Moreover, we cannot rule out the possibility that the inconsistent results may have been due to the small number of subjects who participated in the study. Analyses of the activation of not only BA 2 but also BA 1 and BA 3 will be conducted by increasing the number of subjects in a future study.

In addition, the previous studies have shown that both contralateral and ipsilateral somatosensory cortices were activated by tactile stimulations on the right fingers owing to the bilateral receptive fields of somatosensory cortical neurons^27^ and that the large receptive field size of the somatosensory cortex from the converging tactile inputs led to activation in S2 as well as S1^28^. These indicate that there is a possibility of finding inter- and intra-digit somatotopic representations in ipsilateral BA 2 (or S1 in general). Therefore, in the follow-up studies, we will investigate somatotopic representations of pressure stimulations on the fingers in the bilateral primary and secondary somatosensory cortices to increase our understanding of tactile information processing in the brain.

## Additional Information

**How to cite this article**: Choi, M.-H. *et al*. Somatotopic Map and Inter- and Intra-Digit Distance in Brodmann Area 2 by Pressure Stimulation. *Sci. Rep*. **6**, 30243; doi: 10.1038/srep30243 (2016).

## Figures and Tables

**Table 1:** Group analysis results of the BA 2 activation peak coordinates (anatomical criteria) in MNI coordinates (mm) during pressure stimulation on three joints of each finger.

MNI coordinates (mm)												
	Index finger			Middle finger			Ring finger			Little finger		
	x	y	z	x	y	z	x	y	z	x	y	z
p1	−65	−25	35	−56	−24	34	—	—	—	−51	−30	58
p2	−68	−24	32	−62	−28	44	—	—	—	−48	−25	50
p3	−62	−23	32	−57	−25	44	—	—	—	−50	−24	50

p1: First joint, p2: Second joint, p3: Third joint.

**Table 2:** Inter-digit, intra-digit, and inter/intra-digit distances between BA 2 peak coordinates represented as difference vectors and Euclidean distances (mean ± S.D. through subject analysis).

		Index finger				Middle finger				Ring finger				Little finger			
		Difference vectors			E.D. (mm)	Difference vectors			E.D. (mm)	Difference vectors			E.D. (mm)	Difference vectors			E.D. (mm)
		x	y	z		x	y	z		x	y	z		x	y	z	
Inter-digit	p1	0	0	0	0	−9.1 ± 0.9	−1.9 ± 0.3	1.3 ± 0.8	9.0 ± 1.9	—	—	—	—	−14.8 ± 1.4	−5.4 ± 0.7	−23.3 ± 1.1	**27.3 ± 0.9**
	p2	0	0	0	0	−6.9 ± 2.2	−4.3 ± 0.5	12.2 ± 4.1	14.2 ± 2.4	—	—	—	—	−20.2 ± 1.4	1.2 ± 1.6	−18.5 ± 1.7	26.8 ± 3.5
	p3	0	0	0	0	−5.0 ± 1.7	−2.2 ± 1.4	12.8 ± 2.8	13.7 ± 6.7	—	—	—	—	−12.9 ± 1.5	−1.3 ± 1.6	−18.2 ± 1.4	**22.0 ± 1.6**
Intra-digit	p1	0	0	0	0	0	0	0	0	—	—	—	—	0	0	0	0
	p2	−3.3 ± 1.2	−1.9 ± 0.8	3.3 ± 0.9	4.3 ± 1.0	−6.2 ± 1.3	−4.8 ± 1.1	10.4 ± 0.7	12.2 ± 1.2	—	—	—	—	3.1 ± 0.2	−5.0 ± 1.2	8.3 ± 0.4	9.8 ± 2.1
	p3	−3.3 ± 0.7	−2.8 ± 1.0	−3.4 ± 0.7	7.7 ± 0.7	1.1 ± 0.9	−1.1 ± 1.4	−10.8 ± 1.7	10.2 ± 1.5	—	—	—	—	1.4 ± 1.1	−6.1 ± 0.9	−8.9 ± 0.7	**10.0 ± 0.8**
Inter/intra -digit	p1	0	0	0	0	−9.1 ± 0.9	−1.9 ± 0.3	1.3 ± 0.8	9.0 ± 1.9	—	—	—	—	−14.8 ± 1.4	−5.4 ± 0.7	−23.3 ± 1.1	**27.3 ± 0.9**
	p2	−3.3 ± 1.2	−1.9 ± 0.8	3.3 ± 0.9	4.3 ± 1.0	−3.8 ± 0.9	−3.2 ± 1.4	9.3 ± 0.7	9.9 ± 1.1	—	—	—	—	−17.2 ± 1.1	−0.1 ± 1.0	−15.5 ± 0.8	23.2 ± 2.9
	p3	−3.3 ± 0.7	−2.8 ± 1.0	−3.4 ± 0.7	7.7 ± 0.7	−8.4 ± 1.1	−0.8 ± 0.9	−9.3 ± 1.0	12.0 ± 0.9	—	—	—	—	−15.1 ± 0.9	−1.9 ± 0.8	−15.0 ± 1.0	21.8 ± 3.0

p1: first joint, p2: second joint, p3: third joint.
